# Scraps

**Published:** 1886-12

**Authors:** 


### SCRAPS

PICKED UP BY THE ASSISTANT-EDITOR.

**Cooling Medicine.**—In the diary of a young lady crossing the ocean the following was found: "At eight o'clock in the evening took a pill. At six in the morning passed an iceberg."—*Birmingham Medical Review.*

**Not a Specialist.**—At a public dinner in a rising town a local magnate sitting near the chairman was asked what he thought of a certain sherry. He replied: "I dare say it 's very good; but I 'm not an *accoucheur.*"

**Too Hasty.**—A New York physician boasted at dinner that he had no fear of trichinæ, because he cured his own hams. "Well," said a lady guest to him, "I think it would be preferable to be your ham than your patient." The doctor fled to Germany.

**Poor Babies.**—An advertisement extolling the virtues of a new make of infants' feeding-bottle winds up as follows: "When the baby has done drinking, it must be unscrewed and laid in a cool place, say under a tap."
A writer advises that, "if the baby does not thrive on fresh milk, it should be boiled."

**A Big Fee.**—During the cholera epidemic in Nashville, Tenn., the late Dr. Bowling attended an old blind negro, who eked out an existence by playing the flute at the street corners. He recovered, and, with a heart overflowing with gratitude, he took his flute and sat under the doctor's bedroom window, and played it the whole night long. Of all the large fees he ever received, the doctor said this was the largest.—*American Lancet.*

**Jenner.**—T. C. M., in the *Cincinnatti Lancet-Clinic,* says; "Jenner was much given to versification. On one occasion he sent a brace of ducks with the following lines to one of his patients:

"'I 've despatched, my dear madam, this scrap of a letter,
To say that Miss Dogby is very much better.
A regular doctor no longer she lacks,
And therefore I 've sent her a couple of quacks!'"

**Tact.**—Sir Richard Jebb was once paid three guineas by a nobleman, from whom he had a right to expect five. Sir Richard dropped the coins on the carpet, when a servant picked them up and restored them; three, and only three. Instead of walking off, Sir Richard continued his search on the carpet. "Are all the guineas found?" asked his lordship, looking round. "There must be two still on the floor," was the answer, "for I have only three." The hint, of course, was taken, and the right sum put down.—*Technics.*

**An Argument for Teetotalism.**—As an instance of "the kind of stuff that takes the rustic by storm," the Rev. Dr. Jessopp, in an article in *The Nineteenth Century,* gives this: 'Ah! you shud ha' heerd him a-goin' on last night; him as the teetotal gentlemen from London sent daywn as our depytation. Lor', that were surprisin'!' The speaker was a dear old ranting preacher, a great friend of mine. 'Well, Tack, what did he say?' 'Say! Bless the Lord! he had 'em abaywt Timothy! "You tell me," ses he, "as Paul wrote that ther letter to Timothy, as Timothy was to take to wine bibbing. Nayw, let any man," ses he, "prove to me as Timothy minded what Paul said, and I 'll ha' no more pledges. Ah!" ses he; "I gnaw what Timothy did. He read that there letter, and he says, same as

I shud—What, me take to drinking? I ain't a-going to do it; not for a thaywsand Pauls, I ain't! Timothy war a man, he war,—he took that there letter, and he hull'd it away from him!"'

**Medical Heroism.**—Mr. Lawson Tait, in an address to the Wolverhampton Medical Society, said: "I have all through my professional life felt that those terms of praise about our craft, that it is honourable and self-sacrificing, are entirely deserved. I have even been able to feel many times that we deserved to rank as a noble profession, a term which has been ascribed to us by the highest authority. There are none who exercise the daily functions of medical practice but carry their lives in their hands, and we do it without the exciting aid of trumpet call and cannon smoke; we do it without the stimulus of the bubble ambition, or the speedy and almost painless death from a rifle ball. When we get a duty call from death we get it through the poisoned finger or the lingering pestilence, and we die in weary agony and without the hero's chaplet. Yet there is never a lack in filling the death vacancy. Day by day we are associated with poverty and misery and death, but most of us die in harness, battling to the end with the unhappiness of all around us, and with the poverty which is the lot of ninety per cent. of our profession."—*Edinburgh Medical Journal.*

**The Attractions of Bath.**—In an article headed "Music in Bath," the *Daily Telegraph* says: "'Welcome to Ba-ath, sir,' said Angelo Cyrus Bantam, Esquire, M.C., to the venerable president of the Pickwick Club, just then cast in damages for breach of promise. Bath has a welcome still; by no means confined to unsuccessful defendants. The Queen City of the West keeps its gates wide open, and would like to see many more enter than is the case in our degenerate day. It looks back with fond regret upon the glories of the past—glory attested by its stately streets, its noble squares, and aerial crescents; while deep down in the profoundest conviction of the beautiful place is an assurance that fortune will come back again laden with the good things of old. Why should it not? The healing waters pour forth in volume as great as when they made a new man of Prince Bladud, or as when the Roman conquerors of Britain constructed the baths, which to this day are stately even in ruin. Hot as ever, I believe, are the waters, and as greatly distinguished by the 'flavour of warm flat-irons,' which, according to Mr. John Smauker, signified the presence of 'killibeate.' The unrivalled position of the city is unrivalled still; its temperature remains as equable as of yore; Pump Room, Assembly Rooms, and Theatre Royal stand where they did; the supply of bath-chairs and Oliver's biscuits is equal to any demand; and, unless my information be altogether wrong, the throne of Beau Nash and Angelo Cyrus Bantam, Esquire, is occupied by an affable potentate who only regrets that more persons do not come and place themselves under his benign rule. Both as a social and sanatory institution, Bath is therefore no less 'fit' than heretofore. It resembles a mill running slowly, because having little to grind. Given more customers, and the machinery will work as merrily as ever. Fickle fashion may one day thus turn to its old love. Some, indeed, declare that there are signs even now of a change—that the Bath season is not a mere simulacrum, but a substantial entity, with promise in it of larger development as history more decidedly resolves to repeat itself in this particular regard. Of necessity the revival will have its differences, but human nature is a pretty constant quantity, and while the waters run, visitors may expect to meet Lord Mutanhed, the Honourable Mr. Crushton, the Dowager Lady Snuphanuph, Mrs. Colonel Wugsby, and other deathless types of social humanity. As far as music goes, Bath retains some of its old features."

